# Metastasis‐enhancing protein KITENIN confers temozolomide resistance on glioblastoma with unmethylated MGMT via upregulation of cancer stem cell makers

**DOI:** 10.1002/ctm2.1804

**Published:** 2024-08-08

**Authors:** Eun‐Jung Ahn, Yeong Jin Kim, Md Rashedunnabi Akanda, Se‐Jeong Oh, Tae‐Young Jung, Shin Jung, Jae‐Hyuk Lee, Sung Sun Kim, Yong Yeon Jeong, Hyung‐Ho Ha, Hoon Hyun, Hangun Kim, Joon Haeng Rhee, Kyung Keun Kim, Kyung‐Hwa Lee, Kyung‐Sub Moon

**Affiliations:** ^1^ Department of Neurosurgery Chonnam National University Hwasun Hospital and Medical School Hwasun South Korea; ^2^ Department of Pathology Chonnam National University Hwasun Hospital and Medical School Hwasun South Korea; ^3^ Department of Radiology Chonnam National University Hwasun Hospital and Medical School Hwasun South Korea; ^4^ College of Pharmacy Sunchon National University Sunchon South Korea; ^5^ Department of Biomedical Sciences Chonnam National University Medical School Hwasun South Korea; ^6^ Medical Research Center (MRC) for Immunotherapy of Cancer Chonnam National University Medical School Hwasun South Korea; ^7^ Department of Pharmacology Chonnam National University Medical School Hwasun South Korea; ^8^ BioMedical Sciences Graduate Program (BMSGP) Chonnam National University Hwasun South Korea

Dear Editor,

Temozolomide (TMZ) is a key chemotherapeutic agent for glioblastoma (GBM), but resistance to TMZ and genetic heterogeneity pose significant challenges. Particularly, unmethylated MGMT promoter (*un*methyl‐MGMT) status has been identified as a poor prognostic indicator for the survival of GBM patients.^1^ While MGMT promoter methylation status has been traditionally linked to TMZ resistance, recent research points to additional factors. Cancer stem cells (CSCs) may contribute to chemo‐/radio resistance in GBM by enhancing DNA response mechanisms.^2^ Among CSC markers, aldehyde dehydrogenase 1A1 (ALDH1A1) and CD44 are associated with oncogenic capacity, stemness preservation and therapeutic resistance.^3^, ^4^ KAI1 COOH‐terminal interacting tetraspanin (KITENIN), a glycoprotein implicated in tumour progression and metastasis across various cancers,^5^ has been linked to GBM progression, through epithelial–mesenchymal transition (EMT) and CSC factors.^6^ Current study proposes that KITENIN promotes TMZ resistance by inducing CSC markers CD44 and ALDH1A1 in GBM with *un*methyl‐MGMT.

To investigate the connection between KITENIN and CSC markers in GBM, we analysed human GBM samples and KITENIN‐modulated GBM cells. Immunohistochemistry (IHC) analysis of human GBM samples revealed a significant correlation between high KITENIN expression and increased CSC marker expression (Figure 1A). This observation was corroborated by Western blot analysis, which demonstrated co‐directional expression of ALDH1A1 and KITENIN (Figure S1A). Further investigation using stable KITENIN‐modulated GBM cells showed that KITENIN knockdown in U251 cells led to downregulation of ALDH1A1, CD44 and CD133, while KITENIN overexpression in GL261 cells resulted in their upregulation (Figure 1B). To validate these results, we examined primary GBM cells with varying KITENIN expression levels (Figure 1C,D). Cells with high KITENIN expression (GBM no. 8) exhibited upregulation of both CSC and EMT markers compared to those with low KITENIN expression (GBM no. 6; Figure 1E). This observation was further substantiated through double immunofluorescence staining of GBM paraffin sections (Figure 1F). KITENIN‐*si* treatment significantly reduced the expression of CSC (Figure 1G) and EMT (Figure S1B) markers in primary GBM cells (no. 8) and stable KITENIN‐overexpressed GL261 cells, reinforcing the link between KITENIN and these markers.

**FIGURE 1:**
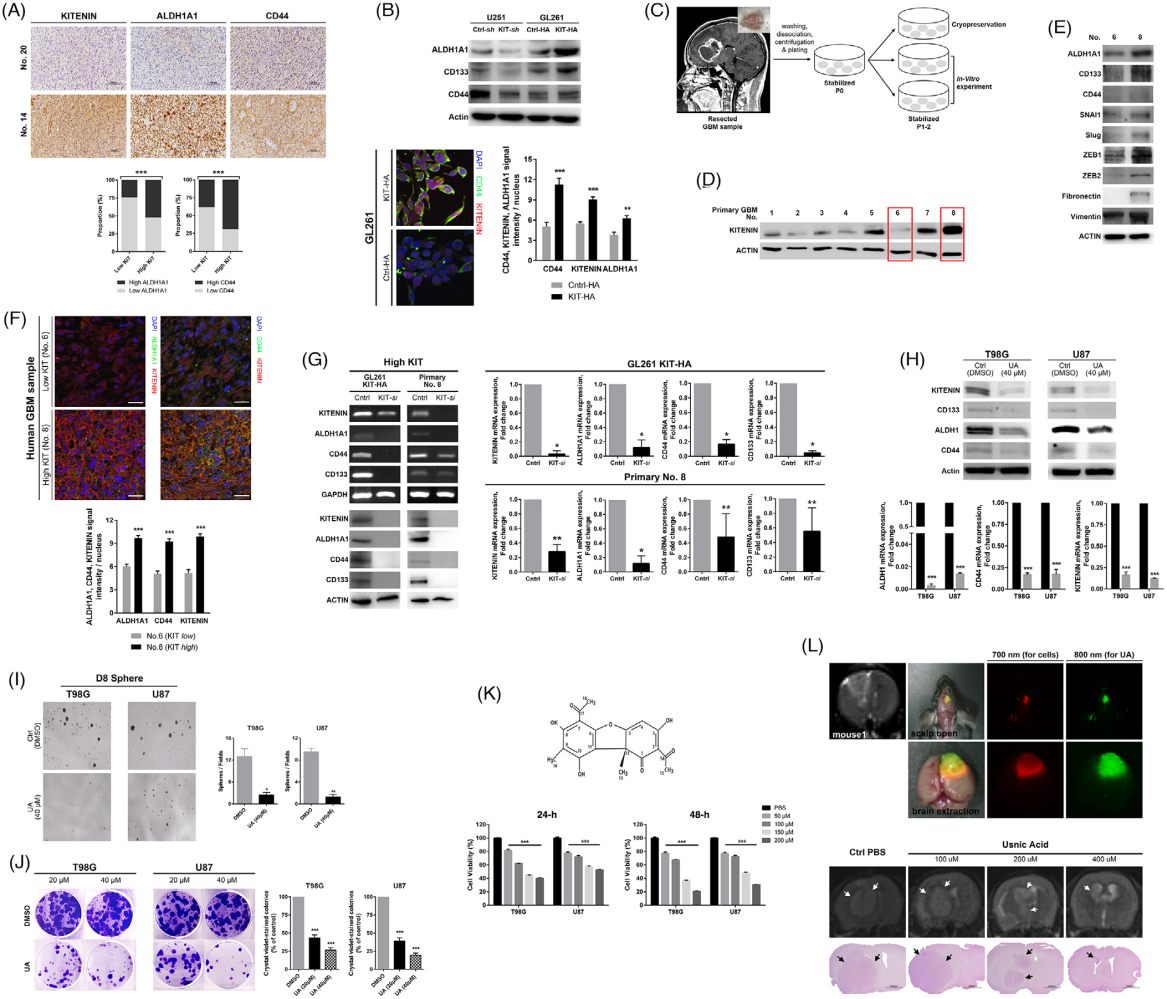
Co‐directional expression of KAI1 COOH‐terminal interacting tetraspanin (KITENIN), aldehyde dehydrogenase 1A1 (ALDH1A1) and CD44 in human glioblastoma (GBM) samples, primary GBM cells and GBM cell lines. (A) Immunohistochemistry (IHC) analysis revealed significantly higher expression of ALDH1A1 and CD44 in patients with high KITENIN expression compared to those with low expression. (B) Western blot analysis showed downregulation and upregulation of ALDH1A1, CD44 and CD133 in KITENIN‐*sh* U251 and KITENIN‐HA GL261 cells, respectively. Immunofluorescence (IF) staining demonstrated elevated co‐expression of KITENIN, CD44 and ALDH1A1 in KITENIN‐HA GL261 cells compared to control cells. (C) A diagram illustrates the establishment of primary GBM cells. (D, E) Primary GBM cells with low (GBM no. 6) or high (no. 8) KITENIN expression were selected from different GBM patients for epithelial–mesenchymal transition (EMT) and cancer stem cell (CSC) markers analysis. Expression of EMT and CSC markers was higher in primary GBM cells with high KITENIN expression than in those with low KITENIN expression. (F) IF staining of pathological samples also demonstrated higher co‐expression of KITENIN/ALDH1A1 and KITENIN/CD44 in samples with high KITENIN expression compared to those with low expression. (G) Western blot, reverse transcription polymerase chain reaction (RT‐PCR) and quantitative real‐time reverse‐transcription PCR (qRT‐PCR) analyses confirmed that higher expression of CSC markers in the stable GL261 cell line and primary GBM cells with high KITENIN expression decreased following KITENIN‐*si* treatment. (H) KITENIN and CSC markers showed reduced expression in T98G and U87 GBM cell lines after usnic acid (UA) treatment, compared to the control (DMSO). (I, J) The sphere and colony‐forming abilities of GBM cells were significantly decreased following UA treatment. (K) Potassium‐usnate (K^+^‐UA) developed for an animal study showed a killing effect on GBM cells similar to the results of UA treatment. (L) Co‐localisation of GBM cells and injected K^+^‐UA was detected by in vivo bioluminescent imaging (*upper panel*). Systemic administration of K^+^‐UA led to the inhibition of tumour growth in an orthotopic mouse GBM model using U87 cells, with mass shrinking on magnetic resonance imaging (MRI) confirmed by histopathology (*lower panel*). Bar graphs show means ± the standard error of the mean (SEM; *p < .05, **p < .01, ***p < .001).

To further explore this connection, we employed the lichen substrate usnic acid (UA), a known KITENIN inhibitor.^7^ UA treatment resulted in significant reduction of KITENIN, ALDH1A1 and CD44 expression (Figure 1H), accompanied by decreased sphere‐/colony‐forming abilities (Figure 1I,J). Moreover, UA inhibited invasion and migration through reducing EMT factors, even at low doses (Figure S2), and decreased cell viability in a dose‐dependent manner (Figure S3). Finally, using an orthotopic GBM model with potassium usnate (K^+^‐UA; Figure 1K), we confirmed the localisation of K^+^‐UA in implanted tumours through in vivo bioluminescent imaging, which led to reduced tumour growth (Figure 1L).

Our clinical analysis revealed that KITENIN's effect on GBM recurrence and survival was contingent on the methylation status of MGMT promoter. In patients with *un*methyl‐MGMT, we observed a significant increase in KITENIN expression in recurrent tumour samples compared to initial samples, along with elevated levels of CD44 and ALDH1A1 (Figure 2A,B). This KITENIN‐CSC axis was found to be a dominant factor in survival analysis of both our GBM cohort and public The Cancer Genome Atlas (TCGA) data. Notably, the impact of KITENIN and CSC markers on survival was only observed in the *un*methyl‐MGMT group, where increased expression of KITENIN and CD44 correlated with poorer progression‐free survival (PFS) and disease‐free survival (DFS; Figure 2C). These survival predictions were further accentuated in combined analysis of these factors (Figure 2D). The differential impact of KITENIN expression based on MGMT methylation status can be explained by the underlying molecular mechanisms. MGMT encodes a DNA repair protein that counteracts the cytotoxic O6‐methylguanine (O6MG) DNA lesions produced by TMZ. Epigenetic silencing of MGMT through promoter methylation enhances TMZ efficacy and prolongs patient survival.^1^ Likewise, the expression of CD44 and ALDH1A1 has been associated with impaired DNA repair in cancer cells, promoting therapeutic resistance.^8^, ^9^, ^10^ In the methyl‐MGMT group, DNA repair capacity is sufficiently compromised to ensure TMZ effectiveness, regardless of KITENIN expression. However, in the *un*methyl‐MGMT group, the preserved DNA repair capacity is modulated by KITENIN expression, influencing TMZ response. This suggests that elevated KITENIN expression contributes to TMZ resistance in GBM patients with *un*methyl‐MGMT, potentially mediated by CSC markers CD44 and ALDH1A1.

**FIGURE 2:**
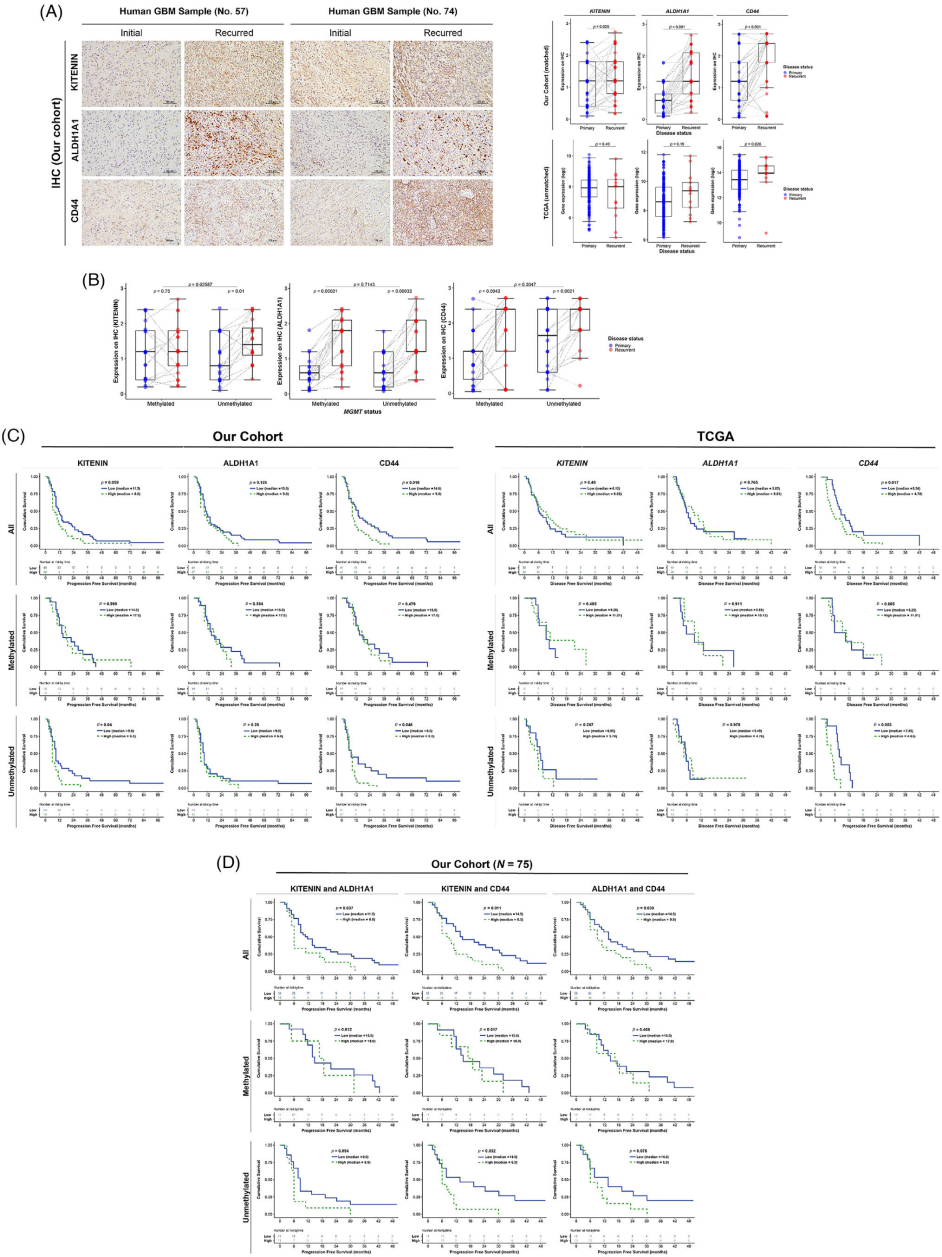
Clinical relevance of co‐directional expression of KAI1 COOH‐terminal interacting tetraspanin (KITENIN), aldehyde dehydrogenase 1A1 (ALDH1A1) and CD44 in *un*methyl‐MGMT glioblastoma (GBM) patients. (A) Representative immunohistochemical (IHC) staining images of KITENIN, ALDH1A1 and CD44 for paired samples (*N* = 40) from GBM patients (initial tumour vs. recurred tumour, our hospital cohort) are shown. In matched samples (*right* upper panel), expression levels of KITENIN (*p *= .025), ALDH1A1 (*p *< .001) and CD44 (*p *< .001) were significantly increased in recurrent tumours compared to initial GBMs, with some cases showing inverse direction. Unmatched The Cancer Genome Atlas (TCGA) data (*right lower panel*) showed significantly higher CD44 gene expression in recurrent tumours (*p *= .026), while ALDH1A1 was higher without statistical significance (*p *= .15). (B) In *un*methyl‐MGMT cases, KITENIN expression increased in recurred tumour (*p *= .01), contrasting with the methyl‐MGMT group (*p *= .75). The difference in KITENIN expression between the methyl‐ and *un*methyl‐MGMT groups was significant (*p* = .02587). Although CD44 expression in recurrent tumours was generally higher than that in initial tumours, this increase was more prominent in the *un*methyl‐MGMT group (*p* = .0021 vs. *p* = .0042 in the methylated group). (C) In our cohort (*N* = 75 GBM cases, IHC analysis, *left panel*), high CD44 expression correlated with short progression‐free survival (PFS; *p* = .018). MGMT methylation status influenced the correlation between KITENIN, ALDH1A1 or CD44 expression and PFS. In the methyl‐MGMT group, high expression of these markers was related to longer median PFS. However, in the *un*methyl‐MGMT group, high KITENIN and CD44 expression was significantly associated with shorter PFS (*p* = .04 and *p* = .048, respectively). The Cancer Genome Atlas (TCGA) data (*N* = 112 GBM cases with MGMT data, *right panel*) showed similar trends for disease‐free survival (DFS), with *CD44* expression significantly related to DFS (*p* = .017), especially in the unmethyl‐MGMT group (*p* = .003). (D) Survival analysis of combined expressions showed that the combination of KITENIN and CD44 provided the most potent survival prediction in GBM patients, particularly in the *un*methyl‐MGMT group (6 months for high expression of both KITENIN and CD44 vs. 14 months for low expression group, *p* = .032).

We established TMZ‐resistant (TMZ‐*R*) GBM cell lines and observed co‐directional expression of KITENIN, CSC and EMT factors compared to parent cells. We developed TMZ‐*R* cells using GBM cell lines with *un*methyl‐MGMT (LN18, T98G, GL261; Figure 3A,B). Strong increases in CD44, ALDH1A1 and KITENIN were observed only in GBM cell lines with *un*methyl‐MGMT (Figure 3C). This was further confirmed in a mouse model using GL261 cells, where tumour cells surviving serial TMZ treatment showed increased expression of KITENIN, CD44 and ALDH1A1 (Figure 3D). Conversely, in TMZ‐*R* cells derived from GBM cell lines with methyl‐MGMT (U87, U251), KITENIN, EMT and CSC markers were decreased (Figure S4).

**FIGURE 3:**
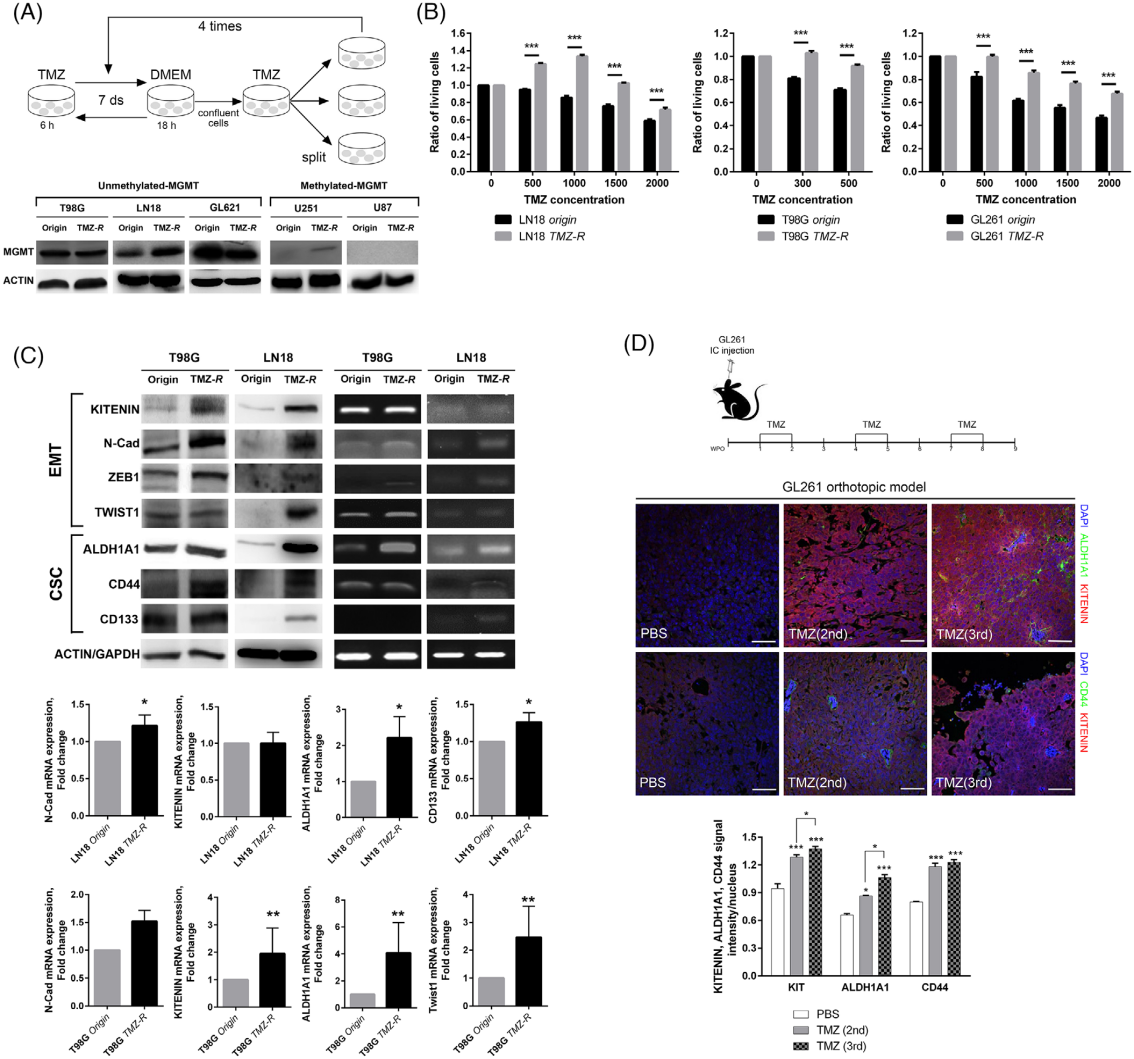
Increased expression of KAI1 COOH‐terminal interacting tetraspanin (KITENIN), epithelial–mesenchymal transition (EMT) and cancer stem cell (CSC) markers in temozolomide‐resistance (TMZ‐*R*) glioblastoma (GBM) cell lines. (A) A diagram illustrates the establishment of various TMZ‐*R* GBM cell lines. T98G, LN18 and GL261 cell lines are classified as *un*methyl‐MGMT GBM cell lines, in contrast to U251 and U87. (B) The established TMZ‐*R* GBM cells demonstrated increased cell viability, as confirmed by 3‐(4,5‐dimethylthiazol‐2‐yl)‐2,5‐diphenyltetrazolium bromide assay following variable doses of TMZ treatment. (C) Western blot analysis revealed higher expression of KITENIN, EMT and CSC markers in TMZ‐*R* GBM cells compared to control. Elevated expression of these markers was also confirmed by reverse transcription polymerase chain reaction (RT‐PCR) and quantitative real‐time reverse‐transcription PCR (qRT‐PCR) analysis. (D) A schematic diagram depicts the TMZ‐treated orthotopic mouse tumour model using GL261 cells. Immunofluorescence (IF) staining of tumour tissue sections showed that co‐expression of KITENIN/aldehyde dehydrogenase 1A1 (ALDH1A1) and KITENIN/CD44 was significantly increased in a treatment‐frequency dependent manner in TMZ‐treated mice, compared to control mice (phosphate‐buffered saline [PBS]). Bar graphs show means ± the standard error of the mean (SEM; *p < .05, **p < .01, ***p < .001).

Additionally, stable KITENIN‐overexpressed GL261 cells demonstrated increased TMZ resistance (Figure S5). In vivo studies demonstrated that mice transplanted with KITENIN‐overexpressed GL261 cells experienced significant tumour regrowth after TMZ treatment (Figure 4A). Immunofluorescence staining of orthotopic GBM tumour sections revealed co‐localisation and high expression of KITENIN/ALDH1A1 and KITENIN/CD44 in mice transplanted with KITENIN‐overexpressed GL261 cells, compared to control mice (Figure 4B). CD133 expression in the KITENIN‐overexpressed GL261 group showed a slight increase compared to the control (Figure 4C). To elucidate the mechanism linking KITENIN with CD44 and ALDH1A1, we focused on the role of AP‐1. KITENIN overexpression increased phosphorylated c‐Jun (p‐c‐Jun) levels, which were abolished by KITENIN‐*si* and DKC1125, a newly identified compound for KITENIN inhibition, along with decreased CD44 and ALDH1A1 levels (Figure 4D). Additionally, inhibition of c‐Fos/AP‐1 by T5244 decreased ALDH1A1 and CD44 expression in KITENIN‐overexpressed GL261 cells (Figure 4E). In GBM cells with *un*methyl‐MGMT, elevated KITENIN expression correlated with increased levels of CD44 and ALDH1A1, potentially mediated through AP‐1 activation, resulting in enhanced TMZ resistance (Figure 4F).

**FIGURE 4:**
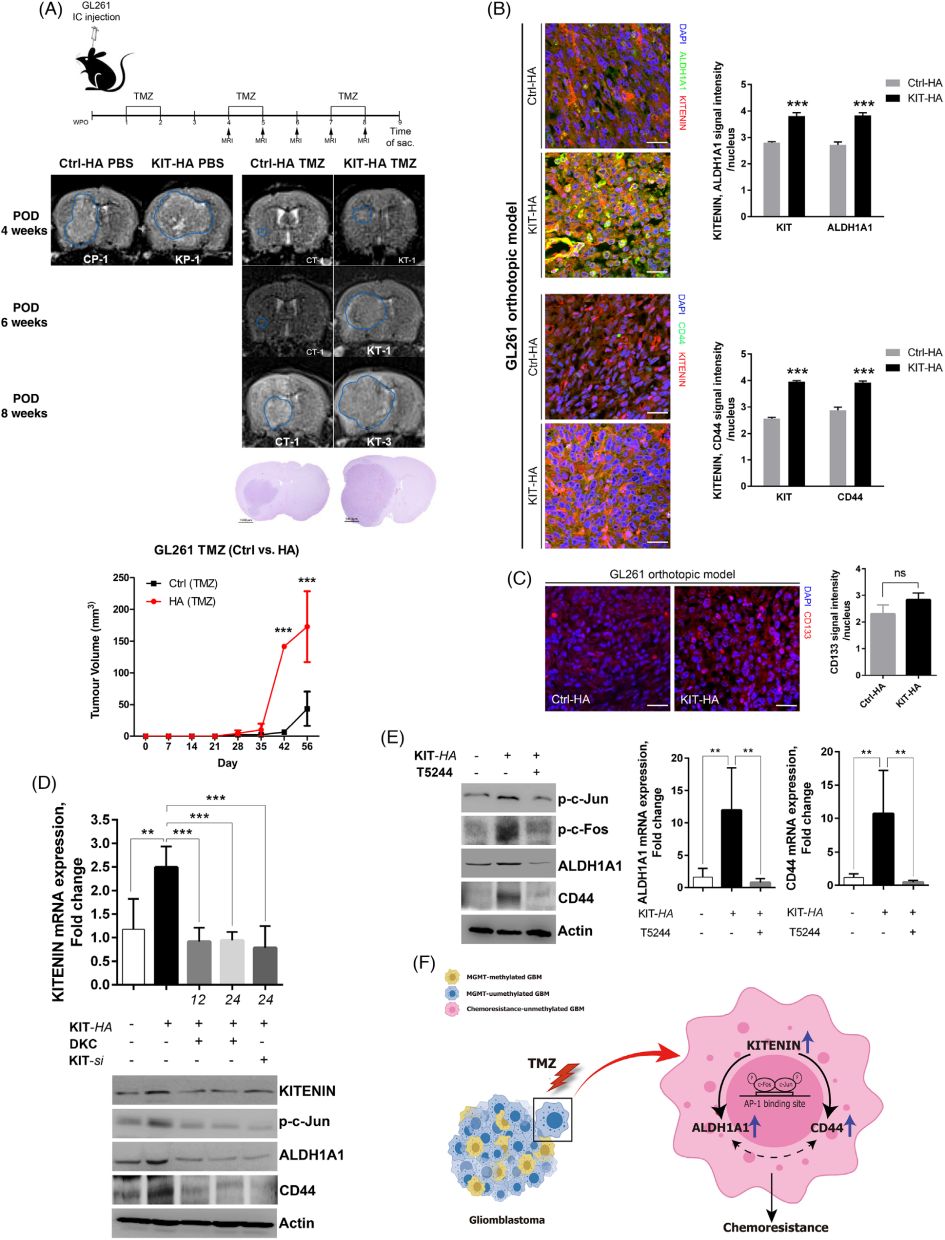
Effects of KAI1 COOH‐terminal interacting tetraspanin (KITENIN) overexpression on tumour progression after temozolomide (TMZ) treatment in an orthotopic mouse glioma model. (A) A schematic diagram illustrates a TMZ‐treated orthotopic mouse glioblastoma (GBM) model using KITENIN‐modulated GL261 cells. Magnetic resonance imaging (MRI) results showed larger tumour growth in mice transplanted with KITENIN‐overexpressed (KIT‐HA) GL261 cells compared to mock‐transfected cells (Ctrl‐HA) without TMZ treatment (treated with phosphate‐buffered saline [PBS]). Under TMZ treatment, initial tumour growth was significantly reduced in both mouse groups, but accelerated tumour re‐growth was more significant in the KIT‐HA group. Histopathological examination at 9 weeks post‐operative day (POD, after three cycles of TMZ treatment) confirmed larger tumours in the KIT‐HA group, with MRI‐based tumour volume comparisons supporting this difference. (B) Immunofluorescence (IF) staining of orthotopic tumour sections showed higher co‐localisation and expression of KITENIN/aldehyde dehydrogenase 1A1 (ALDH1A1) and KITENIN/CD44 in the KIT‐HA group compared to the Ctrl‐HA group. (C) CD133 expression was slightly increased in the KIT‐HA group. (D) Western blot analysis demonstrated that genetic modulation of KITENIN was linked with ALDH1A1 or CD44 levels. Increased KITENIN expression was accompanied by elevation of phosphorylated c‐Jun (p‐c‐Jun), ALDH1A1 and CD44. These elevations were abolished by KITENIN‐*si* and DKC2511 (a KITENIN inhibitor) treatment. (E) Elevated expression of CD44 and ALDH1A1 in KIT‐HA GL261 cells was markedly decreased after treatment with T5244, a c‐Fos/AP‐1 inhibitor, as confirmed by Western blot and quantitative real‐time reverse‐transcription polymerase chain reaction (qRT‐PCR) analyses. (F) A schematic figure illustrates the novel concept of TMZ resistance in *un*methyl‐MGMT GBM, showing the link between KITENIN increase and elevated CD44 and ALDH1A1, possibly through AP‐1 activation. Bar graphs show means ± the standard error of the mean (SEM) (*p < .05, **p < .01, ***p < .001). Ctrl‐HA phosphate‐buffered saline (PBS), Ctrl‐HA group treated with PBS; Ctrl‐HA TMZ, Ctrl‐HA group treated with TMZ; KIT‐HA PBS, KIT‐HA group treated with PBS; KIT‐HA TMZ, KIT‐HA group treated with TMZ.

In conclusion, this study enhances our understanding of the mechanisms driving TMZ resistance in GBM and presents KITENIN as a potential novel therapeutic target, particularly for patients with *un*methyl‐MGMT. These insights may contribute to the development of more effective treatment strategies for this challenging disease.

## AUTHOR CONTRIBUTIONS

Kyung‐Hwa Lee and Kyung‐Sub Moon designed this study. Eun‐Jung Ahn, Yeong Jin Kim, Kyung‐Hwa Lee and Kyung‐Sub Moon drafted the manuscript. Eun‐Jung Ahn and Se‐Jeong Oh performed experiments. Kyung Keun Kim, Hangun Kim, Kyung‐Hwa Lee and Kyung‐Sub Moon performed data analysis. Tae‐Young Jung, Yeong Jin Kim and Shin Jung collected clinical data. Sung Sun Kim, Jae‐Hyuk Lee and Kyung‐Hwa Lee carried out a pathological examination. Md Rashedunnabi Akanda, Shin Jung, Kyung Keun Kim, Joon Haeng Rhee, Hyung‐Ho Ha, Hoon Hyun, Yong Yeon Jeong and Sung Sun Kim assisted with the manuscript preparation and data analysis. Joon Haeng Rhee and Hangun Kim helped with the funding acquisition. Kyung Keun Kim and Joon Haeng Rhee supervised the study. All authors read and approved the final manuscript.

## CONFLICT OF INTEREST STATEMENT

The authors declare no conflicts of interest.

## ETHICS STATEMENT

This study was approved by the Institutional Review Board of Chonnam National University Hwasun Hospital (CNUHH‐2017‐029, CNUHH‐2019‐218). Written informed consent to use clinical data and surgically resected specimens was obtained from all patients or their legal surrogates.

## Supporting information

Supporting Information

## Data Availability

The datasets used and analysed during the current study will be available upon reasonable request.
