# Supplementary material for: Metastasis‐enhancing protein KITENIN confers temozolomide resistance on glioblastoma with unmethylated MGMT via upregulation of cancer stem cell makers
Source: Clin Transl Med. 2024 Aug 8;14(8):e1804. doi: 10.1002/ctm2.1804 (PMC11310266; doi:10.1002/ctm2.1804)
Supplement: Supplementary file 1 — Supporting Information [file CTM2-14-e1804-s001.docx]

**Supplementary Materials**

**Supplementary Figures**

**
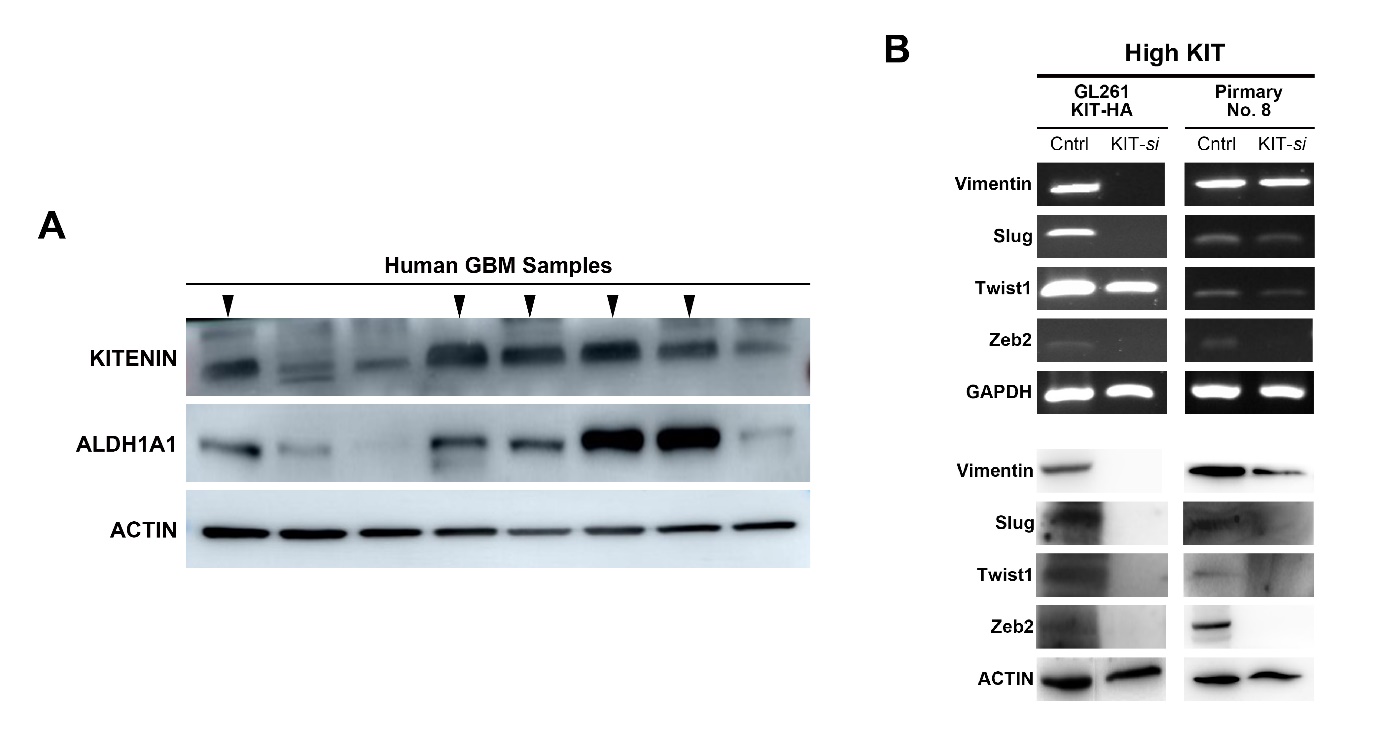
**

**Supplementary Fig. S1. Possible link of KITENIN with ALDH1A1 or EMT markers in GBM.** (A) Western blotting analysis shows that human GBM samples with high KITENIN expression (arrow) exhibited high expression of ALDH1A1. (B) Western blot and RT-PCR analyses confirm that higher expression of EMT markers in the stable GL261 cell line and primary GBM cells with high KITENIN expression decreased following KITENIN-*si* treatment.


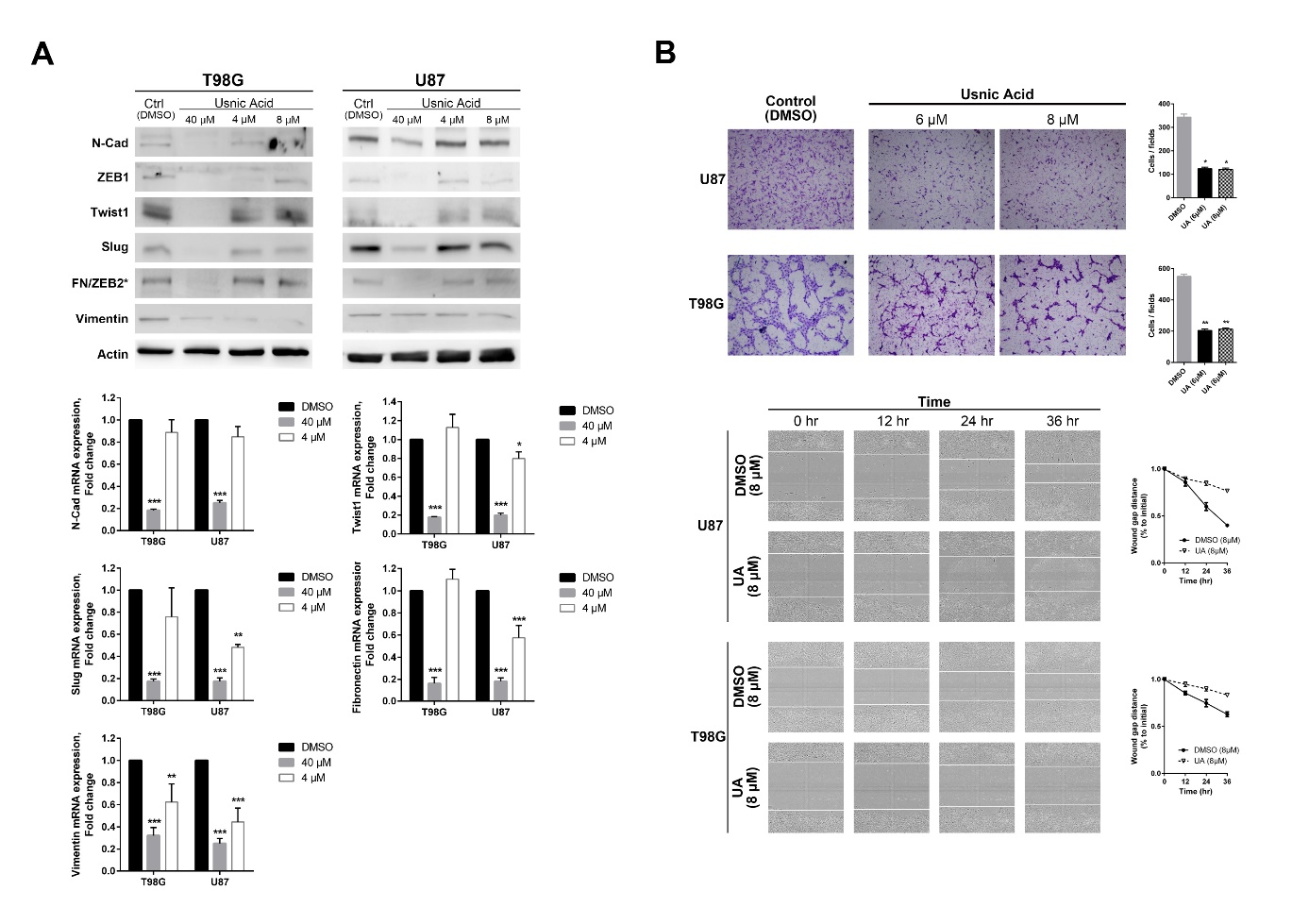


**Supplementary Fig. S2. Effects of usnic acid (UA) on EMT markers and invasion or migration in GBM cells.** (A) UA decreased the expression of EMT markers in T98G and U87 GBM cells as shown by Western blot and qRT-PCR, especially at high UA concentrations. Some EMT factors decreased even at low UA concentrations. (B) UA treatment decreased cell invasion and migration in both GBM cell lines, with this effect observed at relatively low UA concentrations. Bar graphs show means ± the standard error of the mean (SEM) (**P*<0.05, ***P*<0.01, ****P*<0.001).

**
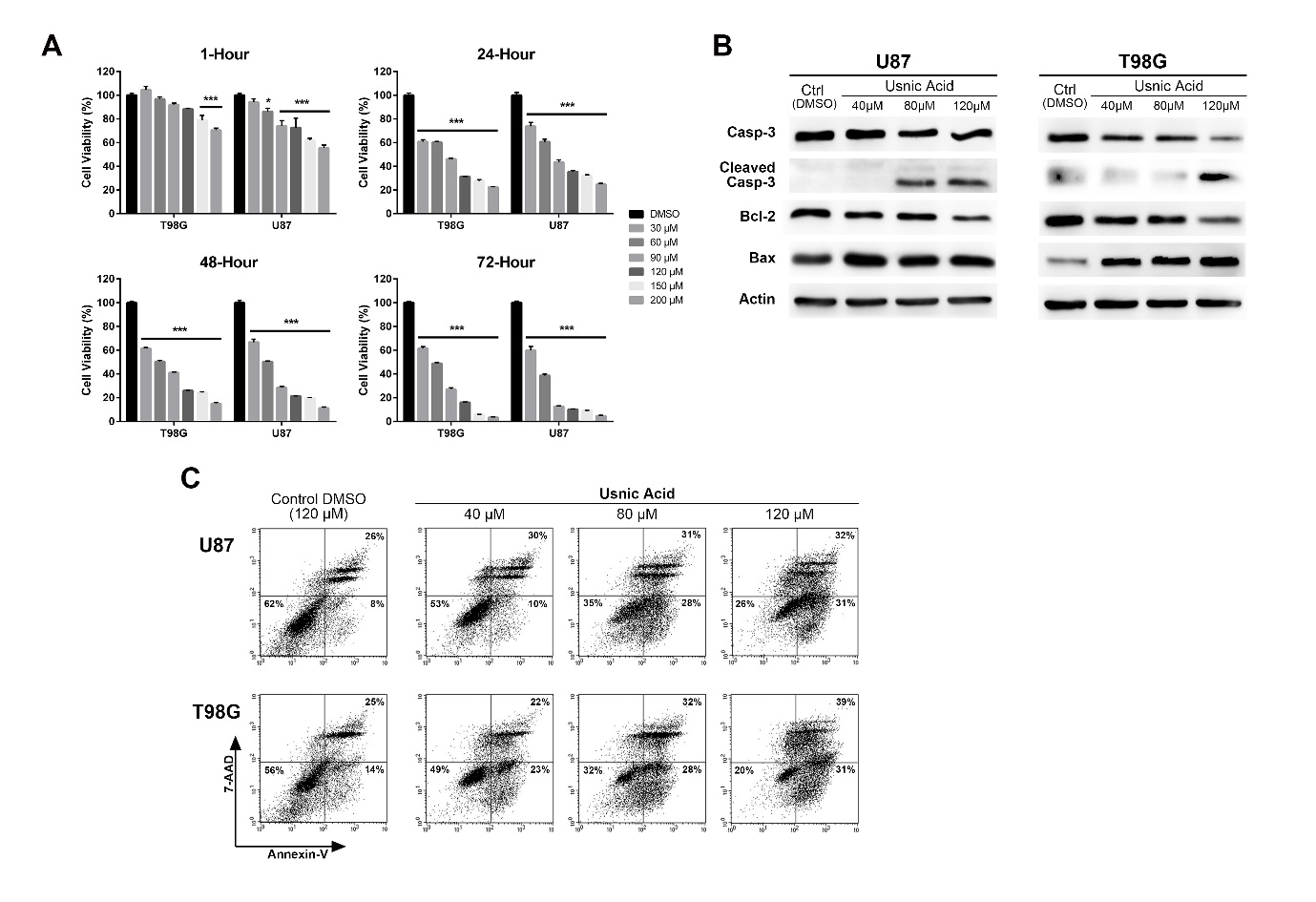
**

**Supplementary Fig. S3. Usnic acid (UA) enhanced apoptosis in GBM cell lines.** (A) MTT assay shows that various concentrations of UA enhanced apoptosis in T98G & U87 cell lines in a dose-dependent manner. The effect of UA on tumor cell viability was observable after 24-h treatment. (B) Molecular-level confirmation through apoptosis marker proteins shows that as the concentration of UA increased, T98G & U87 cells exhibited higher expression of pro-apoptotic cell death markers (cleaved caspase-3, Bax) and lower expression of an anti-apoptosis marker (phospho-Bcl-2) than the control group. (C) Ratio of apoptotic cells was higher after UA treatment in a dose-dependent manner. Bar graphs show means ± the standard error of the mean (SEM) (****P*<0.001).


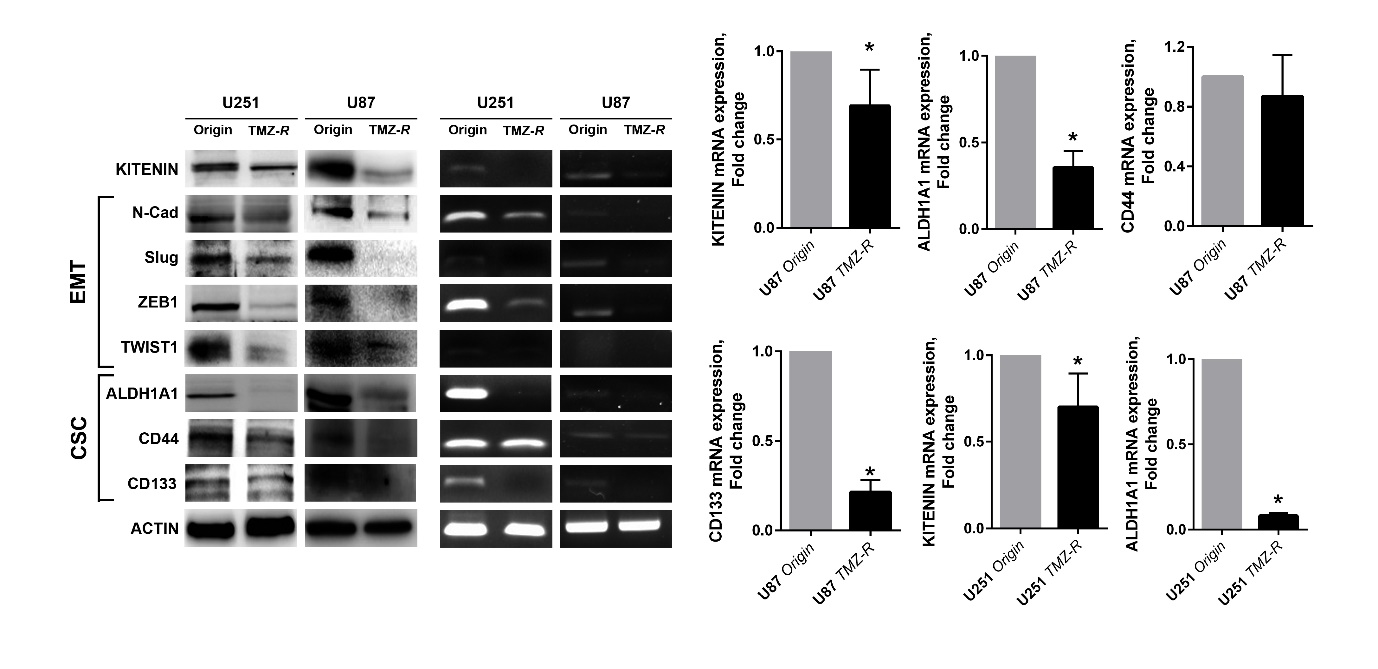


**Supplementary Fig. S4. Opposite KITENIN, EMT, and CSC marker expression patterns in TMZ-resistant (TMZ-*R*) GBM cell lines with methylated MGMT status.** In contrast to GBM cell lines with unmethylated MGMT (T98G, LN18 and GL261), KITENIN, EMT and CSC markers decreased in TMZ-*R* GBM cells with methylated MGMT (U251 & U87) according to Western blot, RT-PCR & qRT-PCR. Bar graphs show means ± the standard error of the mean (SEM) (**P*<0.05).


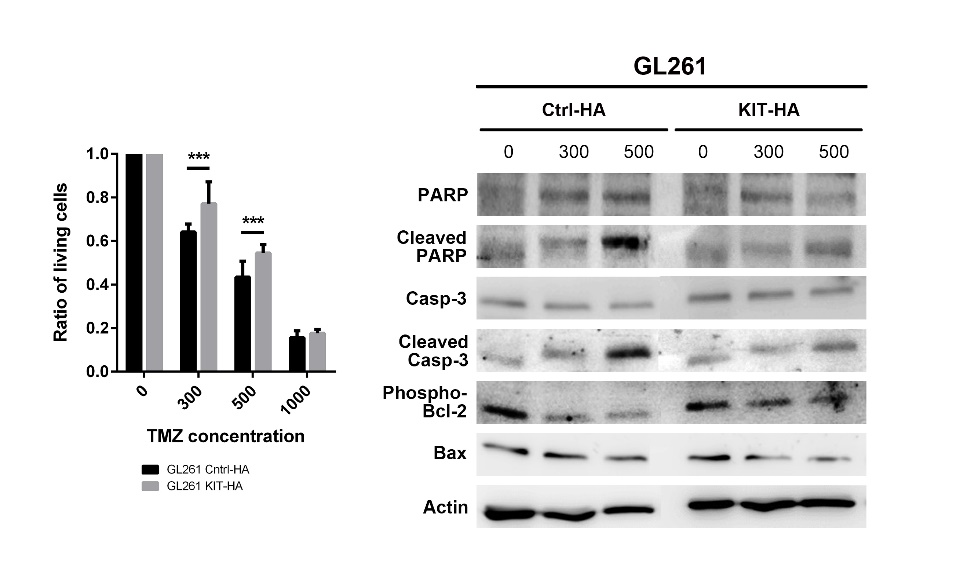


**Supplementary Fig. S5. Genetic modulation of KITENIN affects apoptotic cell death induced by TMZ treatment in GL261 cell line.** (A) MTT assay shows that stable KITENIN-overexpression (KIT-HA) in GL261 cells significantly decreased TMZ sensitivity compared to mock-transfected (Ctrl-HA) GL261 cells (left panel). This result was confirmed by the molecular levels of apoptosis marker proteins. As TMZ concentration increased, KIT-HA GL261 cells showed lower expression of pro-apoptotic cell death markers (cleaved caspase-3, cleaved poly-ADP ribose polymerase (PARP), Bax) and higher expression of anti-apoptosis marker (phospho-Bcl-2) than Ctrl-HA GL261 cells (right panel). Bar graphs show means ± the standard error of the mean (SEM) (****P*<0.001).

**Supplementary Tables**

**Supplementary Table 1: Primers**

| Human genes | Primers sequence (5'-3') | Size (bp) |
| --- | --- | --- |
| *KITENIN* | CGG AAT AAA GAC GGC AGA GG  TGC TCC GAG GTG CCT GTG AT | 152 |
| *CDH2* | TCA GGC GTC TGT AGA GGC TT  ATG CAC ATC CTT CGA TAA GAC TG | 94 |
| *ZEB1* | ATG ACA CAG GAA AGG AAG G  AGC AGT GTC TTG TTG TAG | 158 |
| *ZEB2* | CAA GAG GCG CAA ACA AGC C  GGT TGG CAA TAC CGT CAT CC | 128 |
| *SNAI1* | TCG GAA GCC TAA CTA CAG CGA  AGA TGA GCA TTG GCA GCG AG | 140 |
| *SLUG* | CGA ACT GGA CAC ACA TAC AGT G  CTG AGG ATC TCT GGT TGT GGT | 87 |
| *TWIST-1* | GTC CGC AGT CTT ACG AGG AG  GCT TGA GGG TCT GAA TCT TGC T | 156 |
| *Fibronectin* | CGG TGG CTG TCA GTC AAA G  AAA CCT CGG CTT CCT CCA TAA | 130 |
| *Vimentin* | GAC GCC ATC AAC ACC GAG TT  CTT TGT CGT TGG TTA GCT GGT | 238 |
| *CD133* | GGA CCC ATT GGC ATT CTC  CAG GAC ACA GCA TAG AAT AAT C | 171 |
| *CD44* | TGA ATA TAA CCT GCC GCT TTG  TCC GTC CGA GAG ATG CTG TAG | 73 |
| *ALDH1A1* | TGT CCT ACT CAC CGA TTT G  TCC TTC TTC TAC CTG GCA G | 136 |
| *GAPDH* | AGT TGT CAT GGA TGA CCT TGG C  ATC ACC ATC TTC CAG GAG CGA | 283 |
| Mouse genes | | |
| *KITENIN* | CAA GGA ATA AAG ACG GCA GAG G  TGC TCT GAG GTG CCT GTG AT | 154 |
| *CDH2* | AGC GCA GTC TTA CCG AAG G  TCG CTG CTT TCA TAC TGA ACT TT | 101 |
| *ZEB1* | TGG GAA AGC GTT CAA GTA CAA A  TTG GTT TAC AGA AAG CGG TTC TT | 254 |
| *ZEB2* | AGC CAA GGA ATG CTA CCA A  GGC CCC AGA GCA TCA TAA TC | 143 |
| *SNAI1* | GCC GGA AGC CCA ACT ATA GCG A  TTC AGA GCG CCC AGG CTG AGG TACT | 199 |
| *SLUG* | GCTCCACTCCACTCTCCTTT  CCAGCCCAGAGAACGTAGAA | 244 |
| *TWIST* | CGG GTC ATG GCT AAC GTG  CAG CTT GCC ATC TTG GAG TC | 197 |
| *CD133* | CTC ATG CTT GAG AGA TCA GGC  CGT TGA GGA AGA TGT GCA CC | 222 |
| *CD44* | ATG GCC GCT ACA GTA TCT CC  GCA CAG ATA GCG TTG GGA TG | 178 |
| *ALDH1A1* | GAC AGG CTT TCC AGA TTG GCT C  AAG ACT TTC CAC CAT TGA GTG C | 141 |
| *c-Jun* | TGG GCA CAT CAC CAC TAC AC  TCT GGC TAT GCA GTT CAG CC | 119 |
| *c-Fos* | ATG GCA GAA GGG GCA AAG TAG  GCA ACG CAG ACT TCT CAT CTT CAA G | 172 |
| *GAPDH* | AGT TGT CAT GGA TGA CCT TGG C  ATC ACC ATC TTC CAG GAG CGA | 283 |

**Supplementary Table 2: Antibodies**

| Primary antibodies | Manufacturer | Catalog No. | Dilution | | | |
| --- | --- | --- | --- | --- | --- | --- |
|  |  |  | IF | WB | IHC | FACs |
| KITENIN | Altas | HPA025235 | 1:200 | 1:2500 | 1:200 |  |
| N-cadherin | Abcam | Ab12221 |  | 1:500 |  |  |
| ZEB1 | Bethyl | IHC-00419 |  | 1:500 |  |  |
| ZEB2 | Sigma | HPA003456 |  | 1:50 |  |  |
| SNAI1 | Santa Cruz | Sc-28199 |  | 1:1000 |  |  |
| SLUG | Abcam | Ab38551 |  | 1:500 |  |  |
| TWIST | Abcam | AB50887 |  | 1:1000 |  |  |
| ALDH1A1 | Santa Cruz | Sc-166362 | 1:50 | 1:1000 | 1:50 | 1:50 |
| Vimentin | Abcam | Ab8978 |  | 1:500 |  |  |
| Fibronectin | Abcam | Ab6328 |  | 1:1000 |  |  |
| MGMT | LSBio | LS-C88207 |  | 1:200 |  |  |
| CD44 | Thermo | MA4405 | 1:20 | 1:500 | 1:20 | 1:20 |
| CD133 | Ab Frontier | LF-PA50121 | 1:100 | 1:500 |  |  |
| PARP | Cell signaling | 9542 |  | 1:1000 |  |  |
| Cleaved PARP | Cell signaling | 9541 |  | 1:1000 |  |  |
| Caspase-3 | Cell signaling | 9662 |  | 1:1000 |  |  |
| Cleaved Caspase-3 | Cell signaling | 9664 |  | 1:1000 |  |  |
| Bax | Cell signaling | 2772 |  | 1:1000 |  |  |
| Phospho Bcl-2 | Cell signaling | 2875 |  | 1:1000 |  |  |
| c-Jun | Cell signaling | 9165S |  | 1:1000 |  |  |
| Phospho-c-Jun | Cell signaling | 9261S |  | 1:1000 |  |  |
| Phospho-c-Fos | Cell signaling | 5348T |  | 1:1000 |  |  |
| Actin | Transduction laboratories | 612656 |  | 1:5000 |  |  |

All secondary HRP-linked antibodies for Western Blot detection were purchased from Cell Signaling Technology. All secondary antibodies for immunofluorescence were Alexa Fluor 488/568 purchased from Invitrogen (Thermo Fisher). All secondary antibodies for flow cytometry were Alexa Fluor 647 purchased from Invitrogen (Thermo Fisher). All antibodies were diluted and used according manufacturer’s recommendations

**Methods**

***Cell culture and transfection***

Human GBM cells (U87, LN18, T98G, and U251) were purchased from the American Type Culture Collection (ATCC; Manassas, VA, USA). The mouse GBM cell line, GL261, was kindly provided by Dr. Maciej S. Lesniak (Northwestern University, Chicago, IL, USA). Cells were cultured and transfected according to a previously published protocol and manufacturer’s instructions.^1^

***Cell invasion and migration assay***

Cell invasion and migration assay was performed as described previously.^1^ Cell invasion was examined using 1×10^5^ T98G cells and transwells with chambers separated by filters (8 μm pore size; Corning Inc., Corning, NY, USA) according to the manufacturer’s protocol. T98G cells (1×10^5^) were used. Cells were treated with UA (6 and 8 µM) for 48 h. For the migration assay, 5×10^3^ T98G cells were seeded in 96-well transparent tissue culture plates. Cells were treated with UA (8 µM) for 0, 12, 24, and 36 h. All assays were repeated three times.

***Establishment of TMZ-resistant (TMZ-R) cells***

TMZ-*R* GBM cell lines were established using a previously reported method,^2^ with modifications. Cells were seeded at a density of 2.5×10^4^ cells in 100-cm^2^ dishes, and TMZ was used to treat U87 cells (100 µM); U251, T98G and LN18 cells (300 µM); and GL261 (1000 µM) for 6 h. Then, the medium was replaced with fresh medium and the cells were incubated at 37°C overnight. This treatment was repeated for 7 days. After 7 days, the drug was removed and the medium was replaced with fresh medium. After each treatment, cells were allowed to grow until confluent, and then trypsinized and reseeded at a density of 2.5×10^4^ cells per dish with Dulbecco's Modified Eagle Medium (DMEM) and TMZ for the next round of selection. This treatment was repeated four times.

***Cell viability using MTT assay***

For the 3-[4,5-dimethylthiazol-2-yl]-2,5 diphenyl tetrazolium bromide (MTT) assay, paired parent and TMZ-*R* GBM cells or paired genetically KITENIN-modified GBM cells were seeded on a 96-well plate (5×10^4^ cells/well) and grown overnight. Then, the cells were treated with TMZ (300, 500, 1000, 1500, and 2000 µM) for 48 h. UA in potassium (K^+^-UA) (50, 100, 150, and 200 µM) was used to treat cells for 24 and 48 h. UA in dimethyl sulfoxide (DMSO) (30, 60, 90, 120, 150, and 200 µM) was used to treat cells for 1, 24, 48, and 72 h. The wells were supplemented with MTT (Sigma-Aldrich, Saint Louis, MO, USA) to a final concentration of 5 mg/mL and incubated for 4 h at 37°C. After incubation, l00 µL of DMSO was added to each well. Absorbance was measured at 540 nm using an Infinite 200 PRO NanoQuantMicroplate reader (Tecan Life Sciences, Zurich, Switzerland). Cell viability was calculated as follows: % Cell viability = [Optical density (OD) of the sample/OD of the control] ×100. Half maximal inhibitory concentrations (IC50) were calculated using the SPSS software.

***Annexin V apoptosis assay***

T98G cells were harvested after UA (40, 80, and 120 µM) treatment. After washing with PBS, cells were centrifuged at 1,000 rpm for 3 min. The supernatant was discarded, and pellets were tapped, suspended in binding buffer and incubated for 20 min at RT. We added 5 µL each of Annexin V and 7-aminoactinomycin D (7-AAD), followed by another 15 min of incubation at RT in the dark. Cell's apoptosis levels were determined using flow cytometry (FACSCalibur; BD Biosciences, San Jose, CA, USA).

***Neurosphere culture and colony-forming assay***

T98G cells were plated and grown for 2 days in complete DMEM supplemented with 10% fetal bovine serum (FBS), washed, and re-plated at 10×10^4^ cells/mL in a 60-mm dish. Cells were treated with UA (40 µM) for 48 h. A neurosphere culture assay was performed as described previously ^1^. For the colony-forming assay, T98G cells were grown in 6-well plates at a clonogenic density and treated with UA (20 and 40 µM) for 48 h. After media replace replacement, the cells were incubated for 12 days. Then, the medium was discarded and the colonies were fixed in methyl alcohol and stained with crystal violet (Sigma-Aldrich). Only colonies containing > 25–50 cells were selected for evaluation.

***Near-infrared fluorescence imaging***

Previously described dual-NIR channel FLARE imaging system has been used.^3,4^ In this study, we used 4 mW/cm^2^ of 670 nm excitation light and 11 mW/cm^2^ of 760 nm excitation light, in combination with white light (400–650 nm) at 40,000 lx. Simultaneous color and NIR fluorescence images were captured using custom software at rates up to 15 Hz across a 15 cm diameter. A constant distance of 18 inches was maintained between the imaging system and the surgical field. The exposure time and normalization of images were held constant for each individual experiment.

***Reverse-transcription polymerase chain reaction (RT-PCR) and quantitative real-time PCR (qRT-PCR)***

RT-PCR/qRT-PCR was performed according to the manufacturer’s instructions and previously described method.^1^ Primer sequences used in this study are listed in Table S1.

***Western blot analysis***

Cells and tissue lysis and Western blot analysis were performed as described previously.^1^ The primary and secondary antibodies used in this study are listed in Table S2.

***Immunofluorescence staining***

Genetically paired KITENIN-modified GL261 cells were fixed with 4% formaldehyde, followed by serial alcohol dehydration. After blocking with 2% bovine serum albumin (BSA) for 30 min at RT, cells were permeabilized with 0.1% Triton X-100 for 20 min. Then, the cells were incubated for 16 h at 4°C with primary antibodies. Next, samples were co-incubated with goat anti-mouse IgG secondary antibodies for 1 h at RT. In addition, formalin-fixed paraffin-embedded mouse and human GBM tissues were cut into 3 µm-thick sections and deparaffinized using xylene. Heat-induced antigen retrieval was performed in citrate buffer (pH = 6.0) for 15 min, and 3% hydrogen peroxide was used to inactivate endogenous peroxidase activity. The tissues were incubated for 16 h at 4°C with primary antibodies, and then co-incubated with secondary antibodies for 1 h at RT. Nuclei were counterstained with 300 nM 4’,6-diamidino-2-phenylindole (DAPI) for 20 min, followed by washing with phosphate-buffered saline (PBS). The chamber slides were mounted with anti-fade mounting media and imaged using a laser scanning confocal microscope (FV1000, Olympus Fluoview, Tokyo, Japan). The antibodies used in this experiment are listed in Table S2.

***DKC1125 and AP-1 inhibitor (T5224) treatments***

To investigate the mechanistic link between KITENIN expression and CSC properties, we focused the previous studies proving that KITENIN upregulated AP-1 transcriptional activity,^5,6^ and CD44 and ALDH1A1 were regulated as AP-1 target genes.^7,8^ DKC1125,^9^ a compound newly identified as a KITENIN inhibitor, was kindly provided by Prof. Kyung-Keun Kim (Chonnam National University Medical School, Hwasun, South Korea). T5244 (c-Fos/AP-1 inhibitor) was purchased from the MedChemExpress (Monmouth Junction, NJ, USA) and dissolved in DMSO. To evaluate the effects of T5244 and DKC1125, we estimated the protein and mRNA levels of each target gene. KITENIN-modulated GL261 cells (4×10^5^ cells/2 ml in a 6-well plate) were plated and treated with DKC1125 (1 μM) for 12 and 24 h or T5244 (1 μM) for 48 h.

***Human GBM tissue specimens and clinical data***

Using data from 79 GBM patients who had undergone surgical resection in our hospital, we investigated the clinical relevance of KITENIN, ALDH1A1, and CD44 in GBM. We also included 40 matched samples for recurrent GBM to examine differential expression among these markers. The World Health Organization (WHO) Central Nervous System Classification in 2016 was used for diagnosis.^10^ All patients underwent a surgical resection, followed by concurrent chemoradiotherapy (CCRT) and adjuvant chemotherapy using TMZ. Biopsy cases were not included. Patient demographic data were collected retrospectively. To detect recurrence, we performed serial enhanced magnetic resonance imaging (MRI) at intervals of 1-3 months with and without MR spectroscopy or perfusion study. Based on the Report Assessment for Neuro-Oncology (RANO) criteria,^11^ progression-free survival (PFS) was calculated from the date of initial resection to the date of radiological recurrence, progression, or death. This study was approved by the Institutional Review Board of Chonnam National University Hwasun Hospital (CNUHH-2017-029, CNUHH-2019-218). Written informed consent to use clinical data and surgically resected specimens was obtained from all patients or their legal surrogates.

We carried out immunohistochemistry (IHC) on sectioned samples of human GBM tissue following hematoxylin and eosin (H&E) staining. All H&E and immunostained slides were independently assessed on two separate occasions by experienced pathologists (JHL, SSK, and LKH) who were not privy to the clinical details. The IHC procedures were performed as described in previous research.^1^ The antibodies used in this experiment are listed in Table S2.

***Establishment of GBM patient derived-primary cells***

Fresh GBM tissues were obtained immediately after surgery and were cut into small pieces (≤1 mm). To establish GBM patient-derived primary cells, samples were centrifuged at 400 rpm for 3 min, and the cell pellets were collected. After washing with PBS, cell pellets were cultured in DMEM supplemented with 10% FBS and 1% penicillin-streptomycin. All cells used in these experiments were at early passages (≤ 3).

***Comparative analysis of transcriptomic data from public resources***

To validate results obtained from our cohort, we downloaded mRNA expression profiles and clinical data including recurrence on 166 GBM TCGA samples available on cBioportal for Cancer Genomics (https://www.cbioportal.org/).^12-14^ Gene expression values were log-transformed. We also obtained the O6-methylguanine-DNA-methyltransferase (MGMT) promotor methylation data for patients included in a previous study. Among the 166 patients, both MGMT promoter methylation and expression data were available for 112 patients. Of these, disease-free survival (DFS) analysis was possible in 61 patients.

***Mouse orthotopic intracranial GBM model***

C57BL/6 and nude mice (6-8 weeks old, 15–18 g) were purchased from OrientBio (Seongnam, South Korea). Mice were fed with autoclaved pelleted food and water *ad libitum*. Intracranial injection was performed as described previously.^1^ C57BL/6 were injected with tumor cells [2×10^5^ cells of gene-modulated GL261 cells (Ctrl-HA vs. KIT-HA) in 2 μL of saline] and nude mice were injected with U87 FLUC cells (3×10^5^ in 2.5 µL). TMZ was administrated intraperitoneally (800 μg/100 μl PBS) every 2 days four times, starting from day 7, for a total of 3 cycles at 2-week intervals. K^+^-UA (100, 200, and 400 μM) was administrated intraperitoneally every 2 days three times, starting from day 7, for a total of 2 cycles at 2-week intervals. All animal experiments were performed under the guidelines of the Chonnam National University Medical School Research Institutional Animal Care Committee, and all the experimental protocols were approved by the committee.

***Mouse MRI***

Mouse MRI was performed after 4, 5, 6, and 8 weeks of tumor cell implantation. MRI data were acquired from anesthetized mice using a 3 Tesla MRI scanner (Skyra TIM System; Siemens, Erlangen, Germany). A 3-cm-diameter volume radiofrequency coil (Stark Contrast, Erlangen, Germany) was placed over the mouse’s head. Multi-slice coronal images were acquired using a fast spin echo sequence. T2-weighted images through the brain were produced under the following parameters: 3000 repetitions, 82 ms echo time, field of view of 41 mm, 256 × 176 matrices, slice thickness of 1.0 mm, and 19 slices. The size of intracranial tumors was measured using T2-weighted MRI. Tumor boundaries were manually determined based on the hyperintense region in T2-weighted images. Change in tumor volume over time were derived from these multi-slice MRI data sets for each individual animal. Tumor responses to TMZ treatment were measured before the start of the second cycle of TMZ at 4 weeks post-operatve day (POD) and at 5, 6, 7, and 8 wks POD. At 9 wks POD, the mice were sacrificed for histology examination. Tumor responses to UA were measured on MRI taken at 4 wks POD, immediately before sacrification for histology.

***Statistical analyses***

The Graph Pad Prism v6.00 software for Windows (Graph Pad, La Jolla, CA, USA) was used to analyze in vitro experiments; data are presented as mean ± the standard error of the mean (SEM). Statistical analyses were also performed using the R-4 v4.0.2 software for Mac OS (R Core Team). The collective 2-sided binomial Mann–Whitney–Wilcoxon test was used for comparisons of two groups. The Wilcoxon signed-rank test was performed to compare paired groups using the *wilcox.test()* function in R. Survival analyses were performed using *survival* R package. Kaplan-Meier survival curves were used to estimate patterns of PFS and DFS. Log-rank test [the R function *surv_pvalue()* function] were used to compare Kaplan-Meier curves for survival analysis. Significance was evaluated at a level of *P*<0.05 unless otherwise indicated.

Reference)

1. Lee KH, Ahn EJ, Oh SJ, et al. KITENIN promotes glioma invasiveness and progression, associated with the induction of EMT and stemness markers. *Oncotarget.* 2015;6(5):3240-3253.

2. McDermott M, Eustace AJ, Busschots S, et al. In vitro Development of Chemotherapy and Targeted Therapy Drug-Resistant Cancer Cell Lines: A Practical Guide with Case Studies. *Front Oncol.* 2014;4:40.

3. Ashitate Y, Kim SH, Tanaka E, et al. Two-wavelength near-infrared fluorescence for the quantitation of drug antiplatelet effects in large animal model systems. *J Vasc Surg.* 2012;56(1):171-180.

4. Gioux S, Choi HS, Frangioni JV. Image-guided surgery using invisible near-infrared light: fundamentals of clinical translation. *Mol Imaging.* 2010;9(5):237-255.

5. Bae JA, Yoon S, Park SY, et al. An unconventional KITENIN/ErbB4-mediated downstream signal of EGF upregulates c-Jun and the invasiveness of colorectal cancer cells. *Clin Cancer Res.* 2014;20(15):4115-4128.

6. Kho DH, Bae JA, Lee JH, et al. KITENIN recruits Dishevelled/PKC delta to form a functional complex and controls the migration and invasiveness of colorectal cancer cells. *Gut.* 2009;58(4):509-519.

7. Foster LC, Wiesel P, Huggins GS, et al. Role of activating protein-1 and high mobility group-I(Y) protein in the induction of CD44 gene expression by interleukin-1beta in vascular smooth muscle cells. *Faseb j.* 2000;14(2):368-378.

8. Makia NL, Amunom I, Falkner KC, et al. Activator protein-1 regulation of murine aldehyde dehydrogenase 1a1. *Mol Pharmacol.* 2012;82(4):601-613.

9. Bae JA, Bae WK, Kim SJ, et al. A new KSRP-binding compound suppresses distant metastasis of colorectal cancer by targeting the oncogenic KITENIN complex. *Mol Cancer.* 2021;20(1):78.

10. Louis DN, Ohgaki H, Wiestler OD, Cavenee WK. *WHO Classification of Tumours of the Central Nervous System.* 4th Revised ed. Lyon, France: International Agency for Research on Cancer; 2016.

11. Wen PY, Macdonald DR, Reardon DA, et al. Updated response assessment criteria for high-grade gliomas: response assessment in neuro-oncology working group. *J Clin Oncol.* 2010;28(11):1963-1972.

12. Hoadley KA, Yau C, Hinoue T, et al. Cell-of-Origin Patterns Dominate the Molecular Classification of 10,000 Tumors from 33 Types of Cancer. *Cell.* 2018;173(2):291-304.e296.

13. Cerami E, Gao J, Dogrusoz U, et al. The cBio cancer genomics portal: an open platform for exploring multidimensional cancer genomics data. *Cancer Discov.* 2012;2(5):401-404.

14. Gao J, Aksoy BA, Dogrusoz U, et al. Integrative analysis of complex cancer genomics and clinical profiles using the cBioPortal. *Sci Signal.* 2013;6(269):pl1.
